# Can gadolinium compete with La-Fe-Co-Si in a thermomagnetic generator?

**DOI:** 10.1080/14686996.2021.1957657

**Published:** 2021-08-11

**Authors:** Daniel Dzekan, Anett Diestel, Dietmar Berger, Kornelius Nielsch, Sebastian Fähler

**Affiliations:** a Institute for Metallic Materials, Leibniz IFW Dresden, Dresden, Germany; b Institute for Material Science, TU Dresden, Dresden, Germany

**Keywords:** Energy harvesting, thermomagnetic generator, magnetocaloric materials, thermomagnetic materials, waste heat recovery, 50 Energy Materials, 106 Metallic materials, 203 Magnetics/Spintronics/Superconductors, 206 Energy conversion/transport/storage/recovery

## Abstract

A thermomagnetic generator is a promising technology to harvest low-grade waste heat and convert it into electricity. To make this technology competitive with other technologies for energy harvesting near room temperature, the optimum thermomagnetic material is required. Here we compare the performance of a state of the art thermomagnetic generator using gadolinium and La-Fe-Co-Si as thermomagnetic material, which exhibit strong differences in thermal conductivity and type of magnetic transition. gadolinium is the established benchmark material for magnetocaloric cooling, which follows the reverse energy conversion process as compared to thermomagnetic energy harvesting. Surprisingly, La-Fe-Co-Si outperforms gadolinium in terms of voltage and power output. Our analysis reveals the differences in thermal conductivity are less important than the particular shape of the magnetization curve. In gadolinium an unsymmetrical magnetization curve is responsible for an uncompensated magnetic flux, which results in magnetic stray fields. These stray fields represent an energy barrier in the thermodynamic cycle and reduce the output of the generator. Our detailed experiments and simulations of both, thermomagnetic materials and generator, clearly reveal the importance to minimize magnetic stray fields. This is only possible when using materials with a symmetrical magnetization curve, such as La-Fe-Co-Si.

## Graphical abstract

**Figure:**
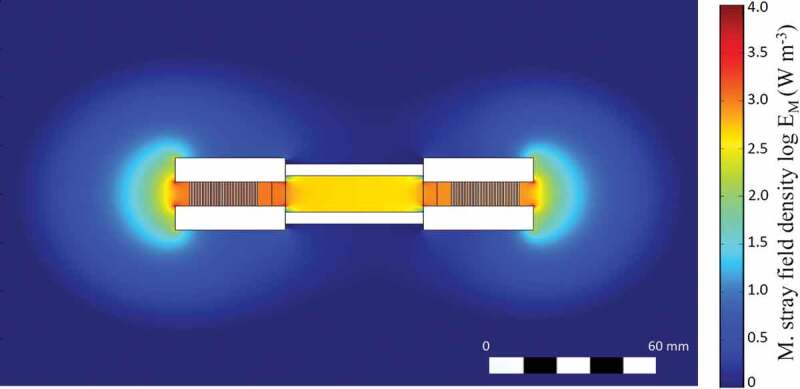

## 1. Introduction

Waste heat is dissipated during almost every type of energy conversion. The recovering of this energy has high potential as the total amount of waste heat sums up to 72% of all electrical energy produced in 2016 [1]. However, 63% of this waste heat has a temperature below 100°C [2]. Only few technologies exist to harvest this low-temperature waste heat and convert it into electricity. The most established applications are thermoelectric generators, but they suffer from a low thermodynamic efficiency or high costs [3,4]. In order to exploit the full potential of low-temperature energy harvesting, other technologies needs to be explored.

One promising alternative is thermomagnetic energy harvesting, which is based on the temperature dependent change of magnetization. This magnetization change is implemented differently in thermomagnetic oscillators [5–7], motors [8] and generators [9–11], but all of them can be described by the same thermodynamic cycle [12]. An overview on the different devices is given by Kishore, Priya [13] and Kitanovski [14].

In this paper we focus on a thermomagnetic generator (TMG) [11], as the only thermomagnetic system, which does not involve any mechanically moving part for the conversion of heat into electrical energy. This conversion is realized by placing a thermomagnetic material inside a magnetic circuit with a permanent magnet as field source. By alternating the temperature of the thermomagnetic material, its magnetization changes, which switches the magnetic flux provided by the permanent magnet. This flux change induces a voltage in a coil winded around the magnetic circuit. First concepts of this working principle were suggested by Tesla [15] and Edison [16] and later Brillouin and Iskenderian calculated a relative efficiency up to 55% [17], which illustrates the high potential of this technology. However, it was only in 2011 when Srivastava et al. realized the first demonstrator [9], even though it had a poor performance due to an unoptimized magnetic circuit. With a more complex magnetic circuit design Christiaanse and Brück were able to reduce magnetic stray fields, which increased the output of their device, although they could not reach the theoretical values [10]. Recently, we developed a TMG prototype with an optimized magnetic field topology, which defines the state of art with respect to induced voltage, output power and efficiency [11]. For the first characterization of the TMG we used a commercial La-Fe-Co-Si alloy [18] as thermomagnetic material, which was originally developed for magnetocaloric cooling at room temperature [19]. Magnetocaloric refrigeration is the reverse energy conversion process of thermomagnetic energy harvesting, where applying a magnetic field on a magnetic material changes its entropy. This entropy change can be utilized to decrease the temperature. For magnetocaloric refrigeration, the ferromagnetic rare earth element gadolinium (Gd) is still the established benchmark material since its high magnetization change around the Curie temperature results in a large entropy and temperature change [20,21]. Therefore it is also considered as thermomagnetic material [8,22–24], because its high magnetization change is also beneficial for thermomagnetic harvesting.

In this paper we compare the performance of Gd within a TMG with the results using La-Fe-Co-Si [11] to examine, if Gd lives up to the expectations for energy harvesting. In both materials the transition is of different order, which has an impact on the performance of the TMG. Almanza et al. predicted with simulations [25], that at low temperature differences the usage of second order materials will result in a lower performance. Using the identical TMG device and similar experimental conditions allow a experimental comparison between both materials also at higher temperature differences. For the comparison, we first describe and highlight the differences of magnetic and thermal properties of both materials. We characterize the magnetic flux and voltage profiles of the generator and measure the electrical power output in dependency of the key process parameters. We explain the differences in voltage and power output by comparing by comparing the magnetic flux change obtained from the material only, by simulations and by experiments within an TMG. In particular, we analyze the difference between experimental and simulated flux change and identify magnetic stray fields as the main cause, since these lead to energy dissipation and a lower performance of the generator. Additionally, we show that the optimum middle temperature in the generator between hot and cold material is not necessarily the Curie temperature of the active material, but depends on the temperature difference. Our comparison identifies the important properties for a high performing thermomagnetic material.

## 2. Experimental setup

**Figure 1.**
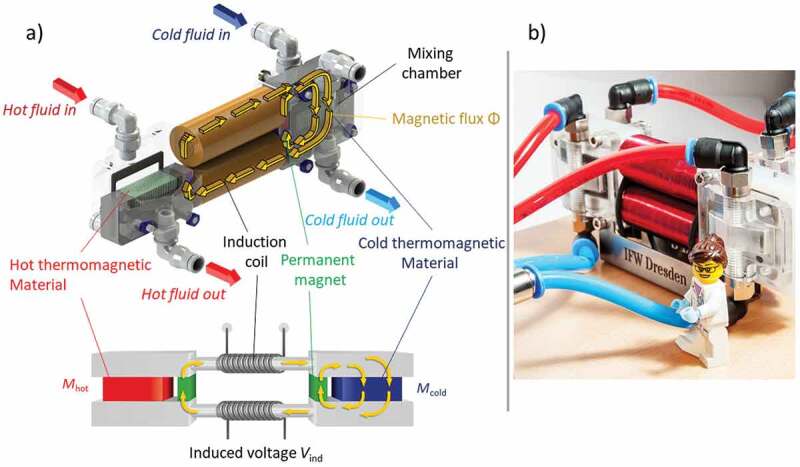
Thermomagnetic generator (TMG) with an optimized magnetic circuit, which allows for a magnetic flux reversal [11]. (a) A technical drawing of the TMG and a scaled version illustrate all components in the design. Two permanent magnets provide a magnetic flux Φ, which is guided over an yoke to the thermomagnetic material. The material is assembled as 25 plates. The plate shape ensure a fast heat exchange with the heat-exchange fluid and a continuous magnetic flux guide. The temperature of the plates alternates due to switching the cold and hot fluid with three-way valves. The plates act as switch for the magnetic flux, which is closed in the cold state (high magnetization $M_{cold}$) and opens in the hot state (low magnetization $M_{hot}$). By the switching, the magnetic flux changes its direction and a voltage is induced within the induction coils. (b) Photo of the TMG with a toy figure as scale

For our experiments we use a thermomagnetic generator (TMG) with an advanced magnetic flux topology. A detailed description is given in our previous work [11]. In this work we used a La-Fe-Co-Si alloy (Calorivac C^®^ from Vacuumschmelze, Germany) as thermomagnetic material [11], which here will be compared with results using Gd. In this section, we introduce the experimental setup of the TMG. In Figure 1(a), a CAD model and a scaled version are shown to illustrate the working principle and all components of the design. Additionally, a photo of the TMG is displayed in Figure 1(b).

In the TMG two Nd-Fe-B permanent magnets generate the magnetic flux Φ. The flux is guided over steel yokes and steel cores with copper coils around, to the thermomagnetic material. On each side, the thermomagnetic material is arranged as a set of 25 La-Fe-Co-Si or Gd plates. For the La-Fe-Co-Si setup, commercial plates were available, whereas the Gd plates were prepared at our lab facility. Therefore, we adjust the thickness of the plates by rolling and cutting to the defined shape (10mm×10mm×0.5mm). After these processes, we restore the magnetic properties by an annealing treatment. In this process, we heat the plates under an argon atmosphere up to 1270°C. We hold the temperature for one hour before we let them cool down by the ambient. The plate shape with a large surface to volume ratio ensures a continuous guide of the magnetic flux within the plates. Additionally, it allows fast heat exchange with the heat-exchange fluid (water with 40% 1,2-propandiol) perpendicular to the plates. Two thermostats pump the fluid through fluid channels between the thermomagnetic material. The channels have the same diameters as the plates. Each chamber has two entrance and exit hose connections for hot and cold fluid, respectively. Four three-way valves ensure that while one side is heated up, the other side is cooled down. The temperature of the fluid is measured before it enters and leaves the mixing chambers immediately by thermoresistors. The fluid flow in the hot and cold liquid is measured before the valves by an impeller flow meter.

The generator is activated when the thermostats cool one side of the plates down to $T_{cold}$ and the other side up to $T_{hot}$. Thereby the magnetic flux closes over the cold side due to the high magnetization $M_{cold}$ of the plates. On the other side, the flux can not pass the hot material with low magnetization $M_{hot}$. With the activation of the valves, hot and cold fluid switch sides, and the thermomagnetic plates change their temperature and magnetization properties. Thus, the thermomagnetic plates act as a thermal switch for the magnetic flux, which changes its direction and closes over the other side. According to Faraday’s law of induction:

(1) $$V_{ind}=-N\frac{d\Phi }{dt}$$

the change of magnetic flux Φ induces a voltage $V_{ind}$ inside the two copper coils. Both have N=1000 windings and are in series to double the voltage. The ends of the coils are connected to a variable resistance chain, which represents the load of the power consumer. Hence, the TMG converts the thermal energy input of the fluids into useful electrical energy.

In order to validate all experimental results with theoretical calculation, we performed finite element simulation of the magnetic field inside the generator. We calculate the field distribution of the magnetic circuit with the COMSOL Multiphysics software (basic module, version 5.5). Furthermore, we performed magnetic measurements of both materials in a Quantum design PPMS using a vibrating sample magnetometer insert.

## 3. Results

### 3.1. Material properties of gadolinium and La-Fe-Co-Si

In this section we describe the functional properties required for thermomagnetic materials and compare the properties of Gd and La-Fe-Co-Si (Calorivac C^®^ from Vacuumschmelze, Germany [18]). A thermomagnetic generator (TMG) requires a temperature-dependent change of magnetization of the thermomagnetic material. A high change of magnetization within a small temperature span results in a large output power and efficiency of the energy conversion. An increase in electrical power is also achievable by a high cycle frequency, which is determined by the time to change the temperature of the thermomagnetic material. Therefore a high thermal diffusivity is beneficial.

Gd is a classical ferromgnet with a hexagonal crystal structure. It orders ferromagnetically during a second order phase transition at the Curie temperature $T_{C}=292$ K [26]. As the magnetic properties of Gd depend on purity and processing [20], we measured the properties of the plates used in the generator.

The La-Fe-Co-Si alloy belongs to the family of $La(Fe_{x}Si_{1-x}{)}_{13}$ with the cubic $NaZn_{13}$ crystal structure [27]. These materials are ferromagnetic at room temperature with a high saturation magnetization due to a high amount of iron. At a transition temperature ($T_{t}$) of 195 K for x=0.88 the material transforms from ferromagnetic state to paramagnetic state within an itinerant electron metamagnetic transition (IEM) [28]. This is a first order transition, which is accompanied by a large volume change of about 1.2% and a thermal hysteresis. In La-Fe-Co-Si, the addition of Co shifts the transition to higher temperatures [19,29] (here $T_{t}=300$K), whereby the type of transition approaches second order. However, the steep change of magnetization still makes a substantial difference compared to the classical ferromagnetic Gd. Accordingly, we compare two types of materials with different behavior of the magnetization around their transition and different shapes of the magnetization curves.

**Figure 2.**
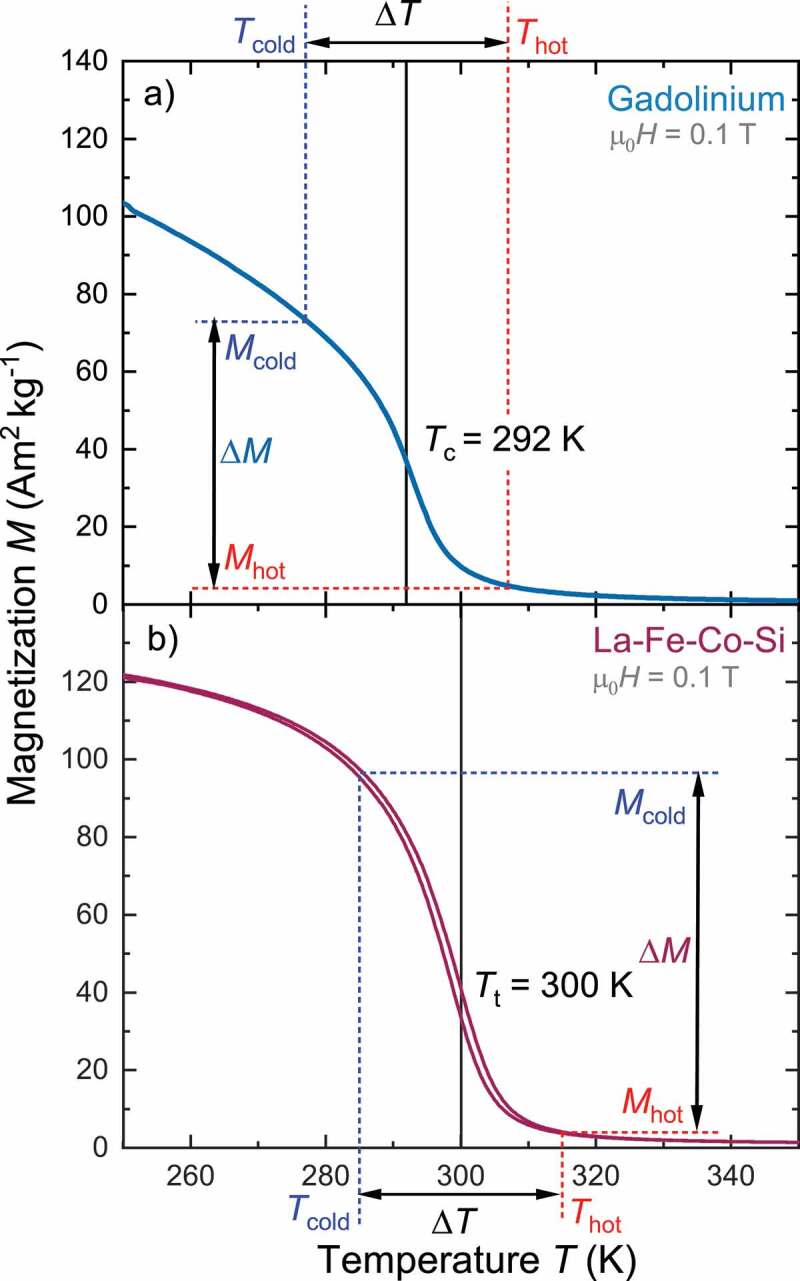
Temperature-dependent magnetization curves for Gadolinium (Gd) and La-Fe-Co-Si. In a thermomagnetic material a temperature change ΔT of e.g. 30 K results in a magnetization change ΔM when the material transforms from the ferromagnetic cold state ($T_{cold}$) with high magnetization ($M_{cold}$) to the paramagnetic hot state ($T_{hot}$) with low magnetization ($M_{hot}$). This is used within a TMG, to switch the magnetic flux. Here we compare two materials with significant different behavior of their magnetization around the transition. (a) Gd changes its magnetization over a broad temperature span and has no hysteresis due to its second order magnetic transition at the Curie temperature $T_{C}=292$ K. (b) In contrast, La-Fe-Co-Si has a steep change of magnetization near the transition temperature $T_{t}=300$ K and a small thermal hysteresis, which are signs for a first order transition. The different characteristic of the M(T)-curves influence the performance of the thermomagnetic material within the TMG. The magnetic field for these measurement is set to 0.1 T, which is similar to the field strength in the TMG

In Figure 2 we show the temperature-dependent magnetization curves at a magnetic field of 0.1 T for Gd (a) and La-Fe-Co-Si (b). This magnetic field is in the same order as the one in the TMG, which we obtain using COMSOL simulations. The temperature difference ΔT results in a magnetization change ΔM when the material goes from the ferromagnetic cold state ($T_{cold}$) with high magnetization ($M_{cold}$) to the paramagnetic hot state ($T_{hot}$) with low magnetization ($M_{hot}$). This is used within a TMG, as it converts thermal energy to electric energy by switching the magnetic flux. With increasing the temperature difference the change of magnetization becomes higher and a larger magnetic flux can be switched. Accordingly, lower ΔT results in a reduced magnetization change and a lower magnetic flux switching. In addition to ΔT, the magnetization change also depends on the middle temperature between $T_{cold}$ and $T_{hot}$: $T_{middle}=\frac{T_{hot}+T_{cold}}{2}$. In this way a magnetization difference is assigned to a middle temperature and temperature difference. We use ΔT and $T_{middle}$ instead of $T_{cold}$ and $T_{hot}$ as a symmetric behaviour is expected commonly for $T_{middle}=T_{C}$ and $T_{t}$.

Within a TMG, the temperature difference is realized by exchanging heat of the thermomagnetic material with the heat exchange fluid. It is desirable that the temperature and the magnetization in the material can change rapidly. By a faster temperature change, the magnetic flux changes more often, which increases the induced voltage. In addition, the output power increases as it is proportional to the higher frequency. Within the same setup, a difference in heat transfer between the fluid and the thermomagnetic material only depends on the material and fluid properties. With constant fluid properties, it is sufficient to describe the problem by the heat diffusion inside the material. The time-dependent temperature change in the magnetic material is given by the heat diffusion equation:

(2) $$\frac{\partial T}{\partial t}=a\nabla^{2}T$$

where t is the time and a the thermal diffusivity $a=\frac{\lambda }{c_{p}\rho }$, which is determined by the heat conductivity λ and volumetric specific heat $c_{p}\rho$. Thereby a larger thermal diffusivity results in a faster temperature change and cycle frequency. In Table 1 the thermal transport properties of both materials are summarized. While all of these properties depend on the temperature, the heat capacity varies strongly at a phase transition and even diverges for a first-order transition due to the latent heat. Here, we averaged the heat capacity for a temperature span of 30 K around the transition. The other properties are taken from literature values at the transition temperature. The value of a for Gd is higher than for La-Fe-Co-Si due to a higher heat conductivity and a lower heat capacity. In particular, the latent heat in La-Fe-Co-Si is the main reason for the lower thermal diffusivity. Accordingly, we expect a faster heat exchange and a higher cycle frequency within the TMG with Gd than using La-Fe-Co-Si.

**Table 1.** The difference in thermal diffusivity a of Gadolinium and La-Fe-Co-Si results in a different heat exchange in the material. The time for changing the temperature in the material determines the cycle frequency. The thermal diffusivity is calculated by the heat conductivity λ, specific heat $c_{p}$ and mass density ρ

	Heat	Specific	Mass	Thermal	Ref.
Material	conductivity λ	heat $c_{p}$	density ρ	diffusivity a	
	(W m^–1^ K^–1^)	(J kg^–1^ K^–1^)	(kg m^–3^)	(m^2^ s^–1^)	
La-Fe-Co-Si	8	750	7800	$5\cdot 10^{-6}$	[18]
Gadolinium	9	600	8900	$9\cdot 10^{-6}$	[34]

### 3.2. From the induced voltage to the experimental flux change

In this section, we measure the magnetic flux change ΔΦ and the induced voltage $V_{ind}$ of the thermomagnetic generator with gadolinium as the active material. Gd is the benchmark material in the magnetocaloric community, due to a significant change of magnetization around the transition at room temperature and its high performance in prototypes [21,30–33]. Therefore it is a promising material for thermomagnetic harvesting and Gd is used in several prototypes [8,22–24]. Accordingly, in this work we probe if Gd also performs well in a thermomagnetic device by comparing the result with our previous characterization of the TMG with a La-Fe-Co-Si alloy [11].

**Figure 3.**
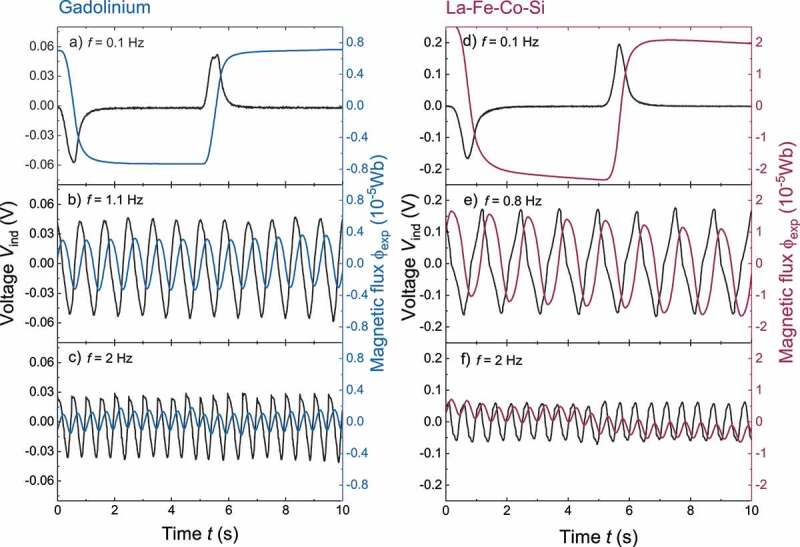
Time-dependent voltage and magnetic flux profiles of the TMG with Gd and La-Fe-Co-Si as active material for different cycle frequencies. Using Faraday’s law of induction, the magnetic flux $\Phi_{exp}$ was calculated from the induced voltage $V_{ind}$. In general, voltage and magnetic flux behave similar in respect to the frequency for both materials. However, their amplitude differs significantly so we use different scales for the y-axis. (a + d) For a low cycle frequency f=0.1 Hz, a voltage is induced only in a short time after switching. During the remaining time, no energy is converted. (b + e) With increasing frequency up to f=1.1 Hz (Gd), respectively f=0.8 Hz (La-Fe-Co-Si) we measured a sinusoidal voltage and flux profile. The amplitude of the voltage and the magnetic flux is reduced compared to the low frequency profiles, but the average voltage over time is significantly higher. (c + f) Further increase of frequency (f=2 Hz) results in lower voltage amplitude and average voltage, since this time is too short for complete heat exchange in the material. All measurements are open-circuit and were performed at a fluid flow of $\dot{V}=0.4$ l min^–1^, a temperature difference ΔT=30 K and a middle temperature identical to the transition temperatures

In a thermomagnetic generator, hot and cold fluid change the temperature of the thermomagnetic material, which leads to a switching of a magnetic flux. By changing the magnetic flux within the coils, a voltage is induced. The frequency of the switching determines the time-dependent voltage profiles [11]. This is shown in Figure 3(a–c) for Gd as active material using three different frequencies. We compare these profiles to the voltage curves of La-Fe-Co-Si (Figure 3(d–f)). All measurements are open-circuit and were performed at a fluid flow of $\dot{V}=0.4$l min^–1^, a temperature difference of ΔT=30K and a middle temperature equal to the transition temperatures. In the TMG with Gd the maximum induced voltage reaches approximately 0.06 V at the frequency f of 0.1 Hz (Figure 3(a)). By using Faraday’s law of induction we obtain the magnetic flux $\Phi_{exp}$(blue line) by integrating the voltage over time. This integration shows that the voltage peak is induced by a magnetic flux change of about 1.4 × 10^–5^ Wb within 1 s. During the same time interval, the induced voltage in La-Fe-Co-Si reaches 0.2 V with a magnetic flux change of about 4.5 × 10^–5^ Wb. We use different scales for both materials due to significantly higher values of $\Delta \Phi_{exp}$ and $V_{ind}$ with La-Fe-Co-Si as active material. By increasing the switching frequency up to 1.1 Hz (Figure 3(b)), we measure a sinusoidal voltage and magnetic flux change with Gd. The induced voltage of approx. 0.05 V and flux change of approx. 0.8 × 10^–5^ Wb is lower compared to the values at the lower frequency. We attribute this to an incomplete heat exchange and thus lower temperature change, which reduces the magnetization change within the Gd plates. However, the average absolute induced voltage is significantly higher than the values at 0.1 Hz. Indeed, we find later that a cycle frequency of 1.1 Hz results in the highest output power. We observe nearly the same behavior for La-Fe-Co-Si (Figure 3(e)), though the values of induced voltage and magnetic flux change are much higher than for Gd. However, the frequency, which results in the maximum output power is obtained at 0.8 Hz. This frequency is slightly lower than for Gd. By increasing frequency up to 2 Hz (Figure 3(c+f)), the time for the temperature change is shortened to the half. Therefore the magnetic flux change and the induced voltage are reduced significantly for both materials. Thereby the average absolute induced voltage is lower as well. Accordingly, a frequency optimization is necessary to find the optimum between a continuous magnetic flux switching and a sufficient time to change the temperature and thus the magnetization of the material.

### 3.3. Electrical output power of the generator

**Figure 4.**
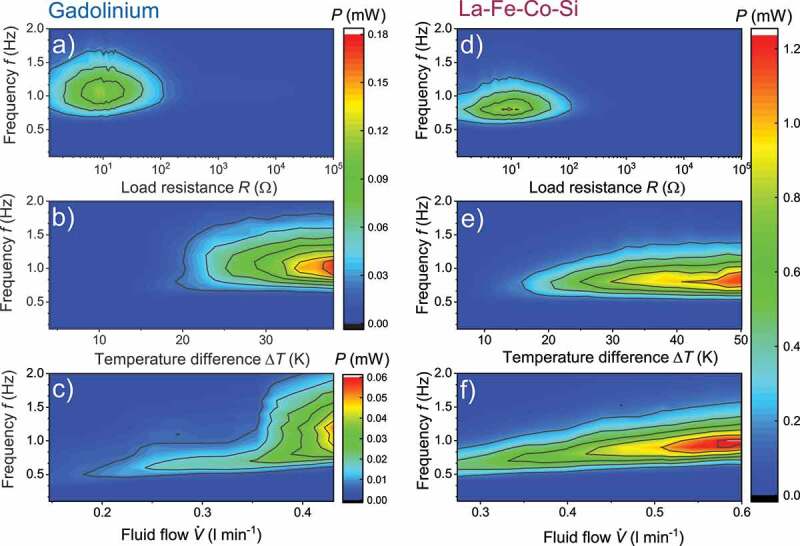
Measured power output of the TMG with Gd (left column) and La-Fe-Co-Si (right column) depending on the key operating parameters. We measured the power output $P=\frac{{\bar{|V_{ind}|}}^{2}}{R}$ with the average induced voltage $\bar{|V_{ind}|}$ and the load resistance R. As $\bar{|V_{ind}|}$ depends on the cycle frequency f, we varied the frequency between 0.1 Hz and 2 Hz to find the optimal f, which results in the highest power output for every key parameter. (a + d) At a constant temperature difference ΔT=30 K, we varied the load resistance. At the optimum, R=9.2 Ω and f=1.1 Hz (Gd) respectively f=0.8 Hz (La-Fe-Co-Si) the highest power was measured. (b + e) With the optimal load resistance we varied the temperature difference ΔT. The power output increases continuously with ΔT, whereby the optimal frequency did not change. (c + f) For ΔT=20 K (Gd) and ΔT=32 K (La-Fe-Co-Si) we varied the fluid flow $\dot{V}$ of the heat exchange fluid. A continuous increase of the cycle frequency results in a higher power output. Different color scales were used due to the significant higher power output obtained for La-Fe-Co-Si

The objective of a TMG is to generate an electric output power, which requires a high current in addition to the induced voltage. In this section, we measure the output power $P=\frac{|\bar{V_{ind}}{|}^{2}}{R}$ by using the average of the induced voltage $|\bar{V_{ind}}|$ and the connected load resistance R in dependence of the key process parameters. For the maximum power, we adjust the load resistance to the internal resistance of the generator. The output power as a function of the load resistance and cycle frequency is displayed in Figure 4 for Gd (a) and La-Fe-Co-Si (d). When the internal resistance is much higher than the load resistance, the current is high, but the induced voltage approaches zero. In contrast, the induced voltage is maximum at a load resistance much larger than the internal one, but then the current is low. In both cases, the output power is minimum. To find the optimal resistance for the maximum power, we use a variable load resistance from 1.15 Ω to 100 kΩ and a constant temperature difference of 30 K.

In our optimization, we find an optimal load resistance of 9.2 Ω for both materials, since this parameter only depends on the design of the generator and not on the material. The optimal load resistance is slightly higher than the DC resistance of both induction coils (7.2 Ω). For Gd, we measure a maximal output power of 0.12 mW at a frequency of 1.1 Hz. This value is significantly lower than the maximal output power reached by the TMG with La-Fe-Co-Si, which is about 0.8 mW at a slightly lower frequency of 0.8 Hz. We keep the optimal load resistance constant in the following measurements.

In the next step, we change the temperature difference ΔT between cold and hot fluid. The middle temperature is fixed to the Curie temperature of Gd (292 K) and the transition temperature of La-Fe-Co-Si (300 K). The temperature difference between cold and hot material determines the change of magnetization and thus the magnetic flux change in the generator. We varied the temperature difference over a broad range and measured the frequency-dependent output power P for Gd (Figure 4(b)) and La-Fe-Co-Si (Figure 4(e)). The power increases continuously with higher temperature differences.

For the TMG with Gd, the highest reachable ΔT in our setup is 36 K without a significant decrease of the fluid flow. Above that, the temperature of the cold fluid is lower than 273 K. At this temperature, the fluid viscosity is much higher and it is more difficult to pump the water-based fluid through the channels between the plates without a drop of the fluid flow. Using La-Fe-Co-Si, a temperature difference up to 50 K can be examined. At the highest ΔT, the power output of the TMG approaches 0.18 mW with Gd and 1.2 mW with La-Fe-Co-Si, which is one order of magnitude difference. When varying ΔT we observe that the optimum frequency for the highest output power remains constant. We attribute this to the heat diffusion, which requires the same time to change the temperature almost independent of ΔT.

In the third step, we measure the influence of the fluid flow $\dot{V}$ on the output power at constant temperature differences ΔT=20 K (Figure 4(c)) for Gd and ΔT=32 K (Figure 4(f)) for La-Fe-Co-Si. Because of the viscosity increase at low temperatures, only a lower ΔT range is accessible for Gd as described before. The output power increases with fluid flow. With faster fluid flow, also the time required for the temperature change is reduced. Thereby two contributions influence the temperature change in the plates: First, the heat exchange between the fluid and the thermomagnetic material. By assuming only heat transfer by conduction, the time for heat exchange depends simply on the heat equation and thus on thermal diffusivity in the thermomagnetic material. Consequently, the higher thermal diffusivity in Gd results in higher frequencies. The second, device-dependent contribution is the time the fluid requires to move through the mixing chamber and the fluid channels between the plates. The temperature of the plates changes only when the fluid passes the end of the plates. With increasing fluid flow, this time is shortened linearly, and the frequency increases in the same manner, as we observe in the experiments. Accordingly, the thermal properties of the thermomagnetic material influence the heat exchange, but the geometry of the TMG design is even more decisive for the cycle frequency.

To sum up this section, we observe a significant lower magnetic flux change with Gd in comparison to La-Fe-Co-Si as thermomagnetic material. This reduction using Gd is about 60%, which results in a decrease of the induced voltage by the same fraction. The cycle frequency reached by the TMG with Gd is slightly higher at the same fluid flow rate. The frequency reaches 1.1 Hz compared to 0.8 Hz with La-Fe-Co-Si. The faster switching of the temperature is attributed to the higher thermal diffusivity. Although the cycle time is shortened, the output power is lower by the factor of 5. The lower power is a result of the reduced magnetic flux change. This is more decisive than the cycle time, as the output power is expected to be square of the flux change [17].

### 3.4. Experimental, materials and simulated magnetic flux change

**Figure 5.**
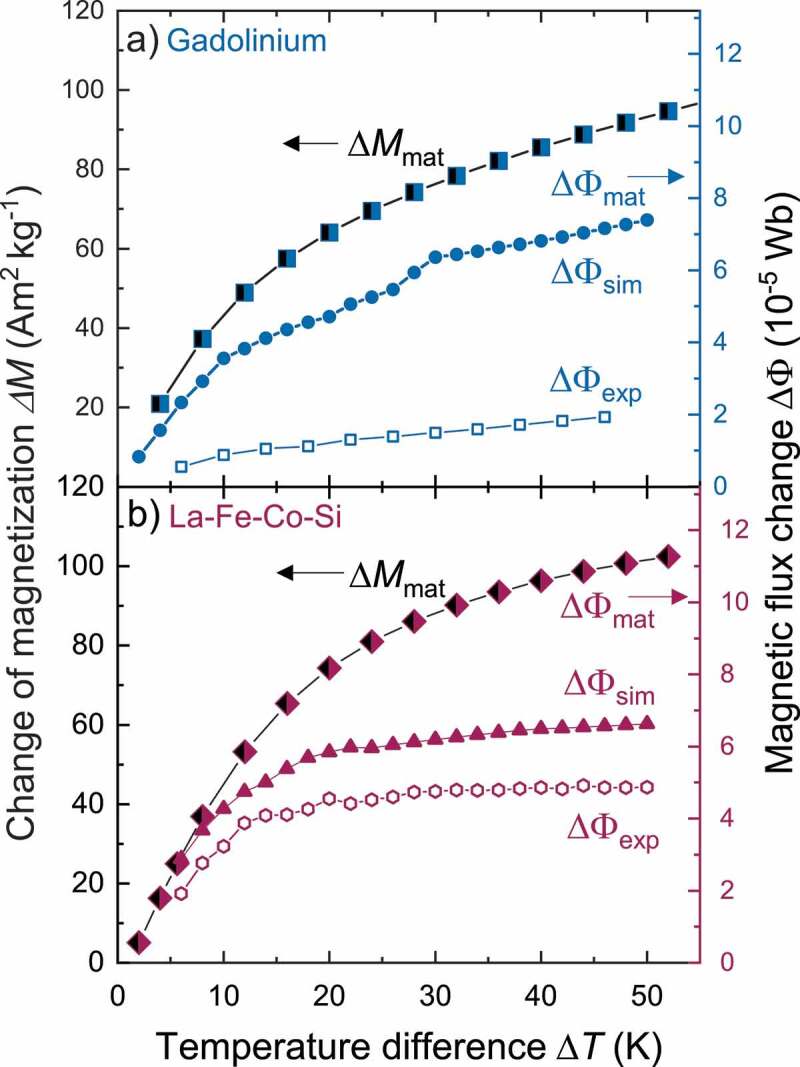
Comparison between materials, experimental and simulated magnetic flux change. For different temperature differences ΔT the magnetization difference ΔM (left axis) is calculated from the magnetization curves M(T) of the materials, whereby the small hysteresis in La-Fe-Co-Si is neglected. With assuming flux conversation in the magnetic circuit of the TMG, the magnetization change is converted with a geometrical factor in an equivalent materials magnetic flux change $\Delta \Phi_{mat}$ (right axis). This allows a direct comparison with the experimental flux change $\Delta \Phi_{exp}$, which was determined from open circuit measurements (3a+d). (a) In our experiments, $\Delta \Phi_{exp}$ of Gd only reaches 25% of the theoretical value. (b) The experimental value for La-Fe-Co-Si reaches about 50% at ΔT=30 K. In addition, we performed finite element simulations. For both materials, this simulated magnetic flux change $\Delta \Phi_{sim}$ reaches values between the theoretical and experimental flux change. In the case of La-Fe-Co-Si the simulated and experimental flux change reach similar values, whereas a large discrepancy of both flux changes is observed for Gd. We explain this difference with the limitations of our simulation model in which only the final states of the temperature change are considered but not intermediate states. As analyzed in Figure 6 magnetic stray fields form during the temperature change process, which result in dissipation. This reduces the magnetic flux change but is not considered in the used equilibrium model

In the previous chapters we already examined experimentally the change of magnetization as the key material property in the TMG. The magnetization change switches the direction of the magnetic flux and thus define the performance of the generator. Now we will derive the flux change directly from the measured magnetization of the thermomagnetic materials and compare it first with the experiments and later with the simulations to explain the lower voltage and power output using Gd. With increasing temperature difference within the TMG, the flux change can be higher due to a larger change of magnetization. In Figure 5 the magnetization difference as a function of the temperature difference is calculated from the temperature-dependent magnetization curves of Gd (a) and La-Fe-Co-Si (b): $\Delta M(\Delta T)=M(T_{t}-\frac{\Delta T}{2})-M(T_{t}+\frac{\Delta T}{2})$. With larger temperature differences, the magnetization change in Gd increases continuously. As ΔM is kind of a derivation, it levels out for large ΔT. This is even more pronounced for La-Fe-Co-Si, as it exhibits a steeper magnetization change around $T_{t}$ and only low changes of magnetization occur at other temperatures. This reflects the first order transformation characteristic. The small transformation hysteresis of the material was considered in the calculations of ΔM by averaging over cooling and heating curves. For both materials, ΔM can be converted in an equivalent material flux change $\Delta \Phi_{mat}$ when the magnetic flux is conserved within the magnetic circuit. As described in [11], the data of the magnetization change represents the flux change (right axis of Figure 5) as well. Since some flux may leave the magnetic circuit, this magnetic flux change represents the upper limit. Next we can compare $\Delta \Phi_{mat}$ with the simulated flux change $\Delta \Phi_{sim}$ from the FEM calculations (for the details of the calculations see [11]). Both flux changes show similar behavior in dependence of ΔT. However, for low temperature differences La-Fe-Co-Si reaches higher values, which agrees with the simulations of Almanza et al. [25]. In contrast to that, the simulated flux change of Gd exceeds slightly the values of La-Fe-Co-Si for higher temperature differences, although the experimental results of Gd give a much lower $\Delta \Phi_{exp}$. To understand this difference, we take a closer look at the experimental flux change in the generator.

**Figure 6.**
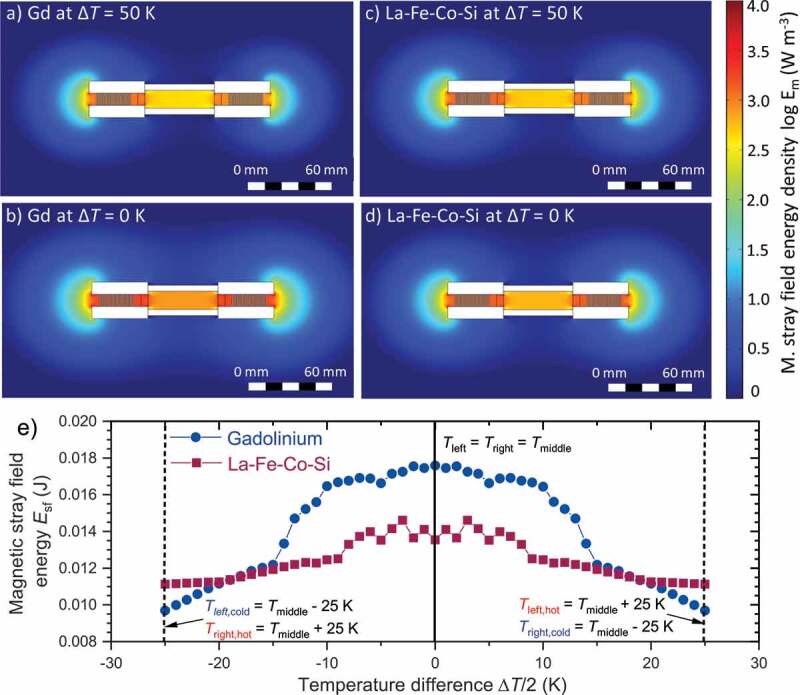
Calculation of the magnetic stray field energy in the TMG using Gd and La-Fe-Co-Si. In case that the magnetic flux of the permanent magnets cannot be guided completely within the magnetic circuit, the magnetic flux has to close over the air which causes magnetic stray fields. The forming of these stray fields represents an energy barrier, which reduces the energy conversion of the TMG. (a) Here, we calculated the magnetic stray field energy density in the TMG using Gd, which is similar to the distribution using La-Fe-Co-Si (c) at the same temperature difference ΔT=50 K. The stray fields occur primarily at the end of the long side of the generator and in between the yokes. In the bottom row the temperature difference is reduced to zero, which means both sides of the generator have the same temperature. The magnetic stray fields increase strongly for Gd (b) and slightly for La-Fe-Co-Si (d). In e) we calculated the strayfield energy by integrating the stray field density over the volume for different temperature differences using Gd and La-Fe-Co-Si. At the x–value equal to zero, both sides of the material have the same temperature, which is the corresponding middle temperature. The left branch of the curves represents the case, that the left side of the generator is cold and the right material hot, whereas the right branch represents the opposite case. During each temperature cycle the temperatures of both sides must cross zero temperature difference, where the maximum strayfield energy has to be overcome. As this energy barrier is higher with Gd, more energy dissipates which reduces the useable magnetic flux

We obtain $\Delta \Phi_{exp}$ as difference between the maximum and minimum flux from the induced voltage profiles by Faraday’s law of induction. While performing these voltage measurements in the same manner as those in Figure 3, we now vary the temperature difference from 6 K to 46 K for Gd. We compare these values with $\Delta \Phi_{exp}$ of the TMG with La-Fe-Co-Si. For La-Fe-Co-Si we observe a similar tendency of $\Delta \Phi_{exp}$ like calculated in the simulation. For small ΔT both properties show a large increase of magnetic flux change. With larger temperature spans, these values approach saturation much faster than the flux change approximation from the magnetization curve. Accordingly, the maximum experimental flux change reaches only 75% of $\Delta \Phi_{sim}$ and 50% of $\Delta \Phi_{mat}$. For Gd, the finite element simulation predicts values of the flux change very close to the material limit. Both flux changes increase slower for Gd with larger temperature spans than observed in La-Fe-Co-Si. However, at higher ΔT the flux changes still increase linearly and thus exceed the flux change in La-Fe-Co-Si. From these results, we expect a higher performance of Gd in the thermomagnetic generator. However, the experimental values of the magnetic flux change are significantly lower than the calculated values. In fact for Gd, $\Delta \Phi_{exp}$ only reaches around 25% of the simulated flux change and 15% of $\Phi_{mat}$. For La-Fe-Co-Si these values are 75% and 50% at a temperature difference of 30 K. This lower magnetic flux change in the TMG with Gd results in a lower induced voltage and thus in a lower output power than expected from the material and simulated flux changes, even for high temperature differences. To explain the difference of the experimental flux change and the simulated one we have to take a closer look at the limitations of our simulation model. In this model we only consider the final states of the heating and cooling process. This means that the temperatures and thus the magnetization are switched completely. However, the temperature and magnetization change needs a certain time and therefore also intermediate states are passed through in the generator. In these intermediate steps magnetic stray fields form, which represent an energy barrier during a thermomagnetic cycle. Accordingly, dissipation occurs, which reduces the magnetic flux change but is not captured in our equilibrium calculations. In the following we describe the forming of these stray fields in the intermediate steps with the key material property: The temperature-dependent change of magnetization and the different shape of M(T) around the transition point.

As shown in Figure 2, the magnetization curves M(T) have different shapes for La-Fe-Co-Si and Gd. This difference originates from the different types of transitions in both materials. In Gd, with a true second-order transition, the magnetization decreases already at temperatures far below $T_{C}$. In contrast, La-Fe-Co-Si retains most of its high magnetization until temperatures close to the transition temperature. At its transition temperature the magnetization drop is much sharper than for Gd. This different behaviour reflects the fact that La-Fe-Co-Si exhibits a transformation with first order characteristics, whereas the transformation of Gd is of second order. The transition temperature for first order materials which is defined as the point of inflection. If we set the middle temperature between cold and hot side in the generator to the transition temperature, the behaviour of hot and cold thermomagnetic material is symmetric in terms of that the increase of magnetization in the colder material is balanced by the magnetization drop in the hotter material. We recently identified this balance as challenge for the design of a TMG, since stray fields represent an energy barrier during a thermodynamic cycle [11]. In Gd we set the middle temperature to the Curie temperature, which results in an highly asymmetric behaviour of the cold and hot side. While on the cold side the magnetization increases continuously, above $T_{C}$ the magnetization is close to zero. This asymmetry results in magnetic stray fields and an energy dissipation, which finally reduces the performance of the TMG. In Figure 6(a+c) we show finite element calculations of the magnetic stray field energy density $w_{sf}$ for a temperature difference of 50 K between the left and right side for Gd and La-Fe-Co-Si, respectively. For Gd (Figure 6(a)) the middle temperature is set as the Curie temperature (292 K). For this temperature difference the cold material has a quite high magnetization and can guide a large amount of magnetic flux, whereby nearly no flux is guided over the hot material with low magnetization. However, the different sides can hardly be distinguished and $w_{sf}$ have nearly a symmetrical distribution. Most of the stray fields form at the long end of the generator and in between the iron yokes. For La-Fe-Co-Si with $T_{middle}$ equal to the transition temperature (300 K) (Figure 6(c)) we observe a similar behaviour with almost the same values of the magnetic stray fields.

In the next step, we set the temperatures of both sides equal to the middle temperature, which is equivalent to a zero temperature difference. By doing so, the materials on both sides have the same reduced magnetization and thus in total can guide less magnetic flux compared to the case of ΔT=50 K. The result is an increase in the magnetic stray field energy density. In particular, this is the case for Gd (Figure 6(b)). The most significant increase is visible inside the TMG and at the edges, but also an expanded range of the stray fields is noticeable. For La-Fe-Co-Si, the stray fields occur in the same areas, although we observe lower values. The lower stray field density results from the higher magnetization at the transition temperature of La-Fe-Co-Si, compared to the magnetization at $T_{C}$ for Gd. Thereby a larger magnetic flux can be guided inside La-Fe-Co-Si at ΔT=0 K.

In Figure 6(e) we calculate the magnetic stray field energy $E_{sf}$ by volumetric integration of the stray field energy density $w_{m}$ for different temperature differences. The black line in the middle represents the case of zero temperature difference, when the material of both sides have the same temperature. At ΔT=0 K, the corresponding magnetic stray field energy density is shown in Figure 6(a+b). At the left dashed line the temperature difference is 50 K, thus the left side in the TMG is cold ($T_{left,cold}$) and the right side hot ($T_{right,hot}$). Therefore this situation corresponds to Figure 6(a+c). The same temperature difference is at the right dashed line, but here the left side is hot ($T_{left,hot}$) and the right side cold ($T_{right,cold}$). Thus when alterning the temperature between both sides, we go from a value at the left branch of the curves to the corresponding value at the right side and back. Each time the maximum at zero temperature difference is passed. Therefore every time when we switch the temperature, the maximum stray field energy has to be overcome. Thermal energy is necessary to overcome this energy barrier, which is significantly higher for using Gd than for La-Fe-Co-Si. Accordingly, more energy gets dissipated and finally, the performance of the TMG is reduced. These calculations consider the complete switching process between hot and cold and are thus more realistic than the simulated flux changes $\Delta \Phi_{sim}$ shown in Figure 5. In other words, the losses by magnetic stray fields explain the difference between $\Delta \Phi_{sim}$ and $\Delta \Phi_{exp}$.

### 3.5. Influence of the middle temperature on the magnetic flux change

**Figure 7.**
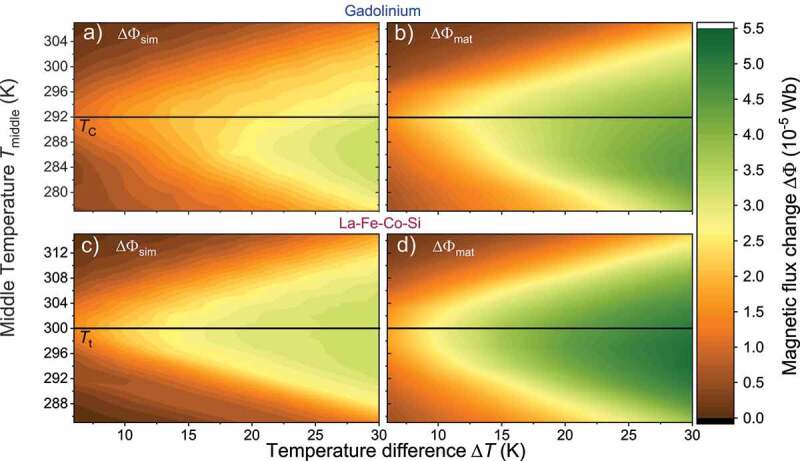
Influence of the middle temperature and temperature difference on the magnetic flux change in the thermomagnetic generator with Gd or La-Fe-Co-Si. Until now we set the middle temperature $T_{middle}=(T_{hot}+T_{cold})/2$ in the TMG equal to the Curie temperature $T_{C}$, respectively the transition temperature $T_{t}$. Here, we calculate the magnetic flux change for different temperature differences ΔT and $T_{middle}$ to determine the best conditions for the highest flux change. The black lines indicate the literature value of the transition temperature. (a + c) The magnetic flux $\Delta \Phi_{sim}$ was calculated with finite element simulation. For Gd (a) we obtain the highest flux change for $T_{C}$ as middle temperature, which decreases with increasing ΔT below $T_{C}$. For La-Fe-Co-Si (c) the flux change is always maximum at the nominal transition temperature. (b + d) Assuming flux conservation in the magnetic circuit of the TMG the change of magnetization ΔM is converted into a magnetic flux change $\Delta \Phi_{mat}$ with a geometrical factor as in Figure 5. We observed a similar behavior of $\Delta \Phi_{mat}$ and $\Delta \Phi_{sim}$ for both used materials. However, the calculated values are higher than the simulated values as they represent the theoretical limit of the flux change given by the material properties. The different behaviour between $\Delta \Phi_{sim}$ and $\Delta \Phi_{mat}$ for Gd reveals that the assumption of flux conservation is not valid. In other words, the asymmetry of the magnetization curve results in unfavourable magnetic stray fields (Figure 6)

As the previous section illustrates the importance of a symmetric magnetization curve to avoid magnetic stray fields, we now examine the influence of the middle temperature $T_{middle}$. For this, we perform further finite element simulations of the magnetic flux change $\Delta \Phi_{sim}$ (see Figure 7). In these simulations we vary $T_{middle}$ and temperature difference ΔT for Gd (Figure 7(a)) and La-Fe-Co-Si (Figure 7(c)). Additionally, in Figure 7(b) we calculate the magnetization difference from the M(T) curves and convert it in an equivalent flux change $\Delta \Phi_{mat}$ as we do in Figure 5. For Gd, we observe a maximum of $\Delta \Phi_{sim}$ at the Curie temperature ($T_{C}=292$ K) for a temperature difference smaller than 16 K. However, for larger ΔT, the magnetic flux change has its maximum at lower middle temperatures. Therefore, the optimal $T_{middle}$ is no longer the same as the Curie temperature of the material. The difference of optimal $T_{middle}$ and $T_{C}$ increases continuously for larger temperature spans. The same is observed for $\Delta \Phi_{mat}$, whereby the flux change reaches higher values, as we describe in Figure 5. In contrast to Gd, in La-Fe-Co-Si the flux changes $\Delta \Phi_{mat}$ and $\Delta \Phi_{sim}$ reach their maximum close to $T_{middle}=T_{t}$.

**Figure 8.**
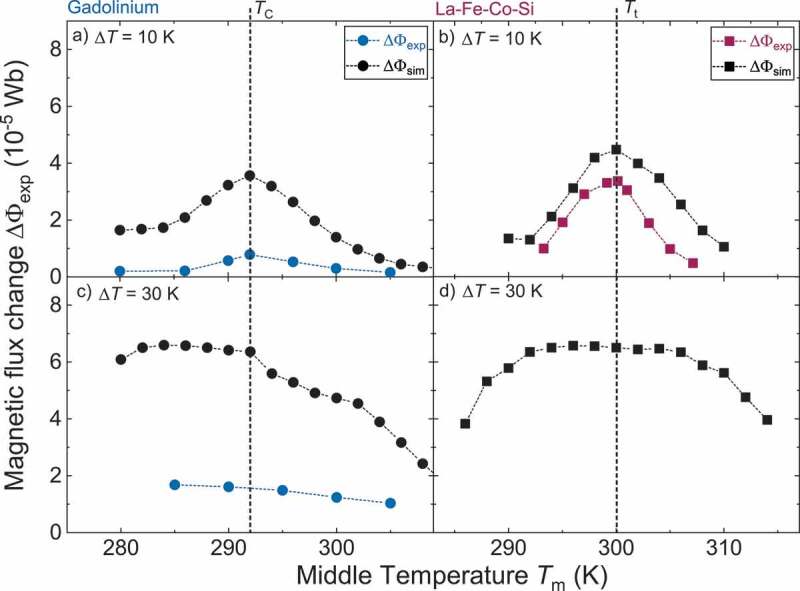
Comparison of simulated and experimental magnetic flux change for different middle temperatures in the thermomagnetic generator with Gd and La-Fe-Co-Si. (a + b) At a low temperature difference ΔT=10 K the maximum of simulated ($\Phi_{sim}$) and experimental ($\Phi_{exp}$) magnetic flux change is observed at a middle temperature $T_{middle}=292\;K=T_{C}$ for Gd and $T_{middle}=300\;K=T_{t}$ for La-Fe-Co-Si, which corresponds to the transition temperatures. (c) For ΔT=30 K the simulated flux change reaches the maximum value at a much lower $T_{middle}=284$ K for Gd. The experimental curve has the same trend, but the absolute values are much lower. For $\Phi_{exp}$ measurements it was not possible to pump the cold liquid at temperatures below 270 K through the generator due to a highly increased viscosity. (d) With La-Fe-Co-Si the broad maximum of simulated magnetic flux change is still around the transition temperature for ΔT=30 K

To validate the calculation results we measure the experimental magnetic flux change $\Delta \Phi_{exp}$, as described in section 3.2. Additionally, we shift the middle temperature $T_{middle}$ to lower and higher temperatures for two constant temperature differences ΔT of 10 K and 30 K. In Figure 8, we plot these curves together with the corresponding simulated magnetic flux change $\Delta \Phi_{sim}$, which represents a sectional view of Figure 7(a) at the relevant temperature differences. For both materials, at ΔT=10 K the experimental flux change follows the trend of the simulated one and a pronounced maximum is observed at $T_{middle}$ equal to $T_{C}$ and $T_{t}$, although with lower values. In particular, this means that $\Delta \Phi_{exp}$ of both materials is low for middle temperatures, which are much higher or lower than $T_{C}$ or $T_{t}$. For this temperature difference the magnetic flux change is maximum if $T_{middle}$ is equal to $T_{C}$ or $T_{t}$ for both materials. For Gd at ΔT=30 K the simulated flux change increases continuously with decreasing $T_{middle}$ until $T_{middle}=285$ K and decreases slightly for lower temperatures. The experimental flux change exhibits a continuous increase for lower middle temperatures as well. However, the absolute values are significantly smaller than in the simulations, as previously discussed.

These results clearly reveal the need of a balanced change of magnetization of the hot and cold side. Thereby the magnetic flux is conserved within the magnetic circuit. This flux conservation avoids magnetic stray fields, which would result in energy loss and a reduction of flux. This is possible when the middle temperature is set to an inflection point, as in the case of La-Fe-Co-Si. Though also Gd exhibits an inflection point slightly below $T_{C}$, the M(T) curve is more symmetrical for a low ΔT. Accordingly in particular for large temperature differences the unsymmetrical magnetization curve around $T_{C}$ leads to an imbalanced increase and reduction of the magnetic flux. In consequence, magnetic stray fields appear, which reduce the performance of the generator significantly.

## 4. Discussion and application

The characterization of different materials within the previously presented thermomagnetic generator (TMG) [11] under similar experimental conditions allows for a direct comparison of Gd and La-Fe-Co-Si for their suitability as thermomagnetic material. In addition, the impact of material properties on the performance of the TMG can be isolated. Gd, as benchmark material for magnetocaloric refrigeration is considered as promising for thermomagnetic harvesting.

In the present work we identify and analyze two decisive properties of thermomagnetic materials: First, a large and symmetric change of magnetization and second a high thermal diffusivity. Furthermore, we probe if Gd also performs well in a thermomagnetic device by comparing the results with our previous characterization of the TMG with a La-Fe-Co-Si alloy [11]. We analyze the time-dependent voltage and magnetic flux profiles of the TMG with both materials as active material at temperature differences of 30 K and the middle temperature equal to the transition temperatures. This revealed a significantly higher average induced voltage and magnetic flux change using La-Fe-Co-Si than Gd. However, the frequency, which results in the maximum output power using Gd, is with 1.1 Hz slightly higher compared to 0.8 Hz for La-Fe-Co-Si. This increase of frequency is much lower than expected from Gd having twice as higher thermal diffusivity [34] compared to La-Fe-Co-Si [19]. The reduction of magnetic flux is around 60% with Gd in comparison to La-Fe-Co-Si, which results in a decrease of the induced voltage by the same magnitude. The faster switching of the temperature is attributed to the higher heat conductivity, respectively temperature diffusivity in Gd. Although the cycle time is shortened, the output power is lower by a factor of 5 due to the reduced magnetic flux change, which is expected to be square of the flux change [17].

We found that the unsymmetrical magnetization curve of Gd leads to an uncompensated magnetic flux in the TMG. Additionally we observed, that the used $T_{middle}$ in the TMG is crucial for the magnetic flux within the system. If $T_{middle}$ is identical to the respective transition temperature of the used thermomagnetic material, the results for Gd and La-Fe-Co-Si are different. Using Gd as active material, optimal values of the magnetic flux were found for $T_{middle}$ below $T_{C}$ and not when $T_{middle}$ is equal to $T_{C}$. The reason for this is that the M(T) curve of Gd is more symmetrically for a low temperature. In particular for large temperature differences the unsymmetrical magnetization curve leads to an imbalanced increase and reduction of the magnetic flux. In consequence, magnetic stray fields occur, which reduce the performance of the generator significantly. Accordingly, these results clearly reveal the need of a balanced change of magnetization of the hot and cold side, whereby magnetic flux is conserved within the magnetic circuit which avoids stray field. As any efficient TMG design must keep the magnetic flux within the system and avoid stray fields, a balanced magnetisation change is of general importance. This aspect is not considered in previous works [12,25] where only the material, but not the system is analyzed.

## 5. Conclusions

Gadolinium has been used for many years in the magnetocaloric community, and today it is still the benchmark material in prototypes due to its high performance [21]. Consequently, it also has been considered as thermomagnetic material [8,22–24] since both methods use the high change of the temperature-dependent magnetization around the ferro- to paramagnetic transition.

In this work, we compared the performance of Gd and La-Fe-Co-Si in the identical state of the art TMG. In this comparison, La-Fe-Co-Si outperformed Gd clearly in terms of induced voltage and output power, although a slightly higher cycle frequency was possible using Gd. As reason for the lower performance of the TMG with Gd we identified the unsymmetrical shape of the temperature-dependent magnetization curve in this second order material. Thereby the decrease of magnetization on the hot side above the transition temperature is not compensated by the same increase of magnetization on the cold side below the transition temperature. This balance is necessary for flux compensation in the magnetic circuit. The imbalance using Gd leads to an uncompensated magnetic flux, which results in magnetic stray fields. These stray fields are an energy barrier during the thermodynamic cycle and reduce significantly the performance of the TMG. Furthermore, we also observed, that the Curie temperature is not the optimal middle temperature $T_{middle}$ between the cold and hot side, but a lower temperature is better. Both aspect clearly demonstrate that it is essential to examine and optimize both together, the functional material and the system. However, this is a general technological challenge for the application of all new functional materials.

In addition to the low performance of the TMG with Gd, this material is expensive [35] and susceptible to corrosion [36]. Therefore we can not recommend the usage of Gd in a TMG, although it has a better mechanical stability and thus it may be useful in particular generator designs. To sum up, we found La-Fe-Co-Si as the better material, which is more suited as benchmark material for thermomagnetic energy harvesting than Gd. For the future developments, we suggest to focus on thermomagnetic materials with a high symmetric shape of the magnetization curve.

## Data Availability

The data that support the findings of this study are available from the corresponding author, D.D., upon reasonable request.
